# Exploring altered bovine sperm trajectories by sperm tracking in unconfined conditions

**DOI:** 10.3389/fvets.2024.1358440

**Published:** 2024-04-02

**Authors:** Luigi Fausto Canonico, Claudia De Clemente, Margarida Fardilha, Ana Filipa Ferreira, Maria Isabella Maremonti, David Dannhauser, Filippo Causa, Paolo Antonio Netti

**Affiliations:** ^1^Interdisciplinary Research Centre on Biomaterials (CRIB) and Dipartimento di Ingegneria Chimica, Dei Materiali e Della Produzione Industriale, University of Naples “Federico II”, Naples, Italy; ^2^Laboratory of Signal Transduction, Institute for Biomedicine-iBiMED, Medical Sciences Department, University of Aveiro, Aveiro, Portugal

**Keywords:** sperm motility, unconfined motion, sperm tracking, sperm classification, swimming patterns

## Abstract

Mammalian sperm motility is getting more relevant due to rising infertility rates worldwide, generating the need to improve conventional analysis and diagnostic approaches. Nowadays, computer assisted sperm analysis (CASA) technologies represent a popular alternative to manual examination which is generally performed by observing sperm motility in very confined geometries. However, under physiological conditions, sperm describe three-dimensional motility patterns which are not well reconstructed by the limited depth of standard acquisition chambers. Therefore, affordable and more versatile alternatives are needed. Here, a motility analysis in unconfined conditions is proposed. In details, the analysis is characterized by a significant longer duration -with respect to conventional systems- with the aim to observe eventually altered motility patterns. Brightfield acquisition in rectangular glass capillaries captured frozen–thawed bovine spermatozoa which were analyzed by means of a self-written tracking routine and classified in sub-populations, based on their curvilinear velocity. To test the versatility of our approach, cypermethrin -a commonly used pesticides- known to be responsible for changes in sperm motility was employed, assessing its effect at three different time-steps. Experimental results showed that such drug induces an increase in sperm velocity and progressiveness as well as circular pattern formation, likely independent of wall interactions. Moreover, this resulted in a redistribution of sperm with the rapid class declining in number with time, but still showing an overall velocity increase. The flexibility of the approach permits parameter modifications with the experimental needs, allowing us to conduct a comprehensive examination of sperm motility. This adaptability facilitated data acquisition which can be computed at different frame rates, extended time periods, and within deeper observation chambers. The suggested approach for sperm analysis exhibits potential as a valuable augmentation to current diagnostic instruments.

## 1 Introduction

Nowadays, the growing demand for milk and dairy products is driving producers to increase livestock productivity to meet market demands. Consequently, there is an increasing reliance on artificial insemination techniques, particularly concerning cows and bulls. Insemination can be regarded as one of the oldest biotechnological methods that has had a significant impact on animal production globally (1–3). In addition, sperm analysis is a crucial step in both diagnosis and treatment of human male infertility. During the last decades, infertility represented a problem affecting more than 35% of couples worldwide, a large part of which is attributable to men reproductive problems (4, 5). For this reason, semen quality evaluation is pivotal to increase the chances of a good fertilization outcome (6). In general, it has been demonstrated that many factors could influence male fertility including anatomical abnormalities, environmental factors, lifestyle and exposure to toxicants and pesticides (7–11). The latter are largely employed in agriculture and represent a high-risk factor for humans and animals by either direct exposure or remotely through ingestion of cereals, fruits, and animal products (12). For example, Cypermethrin (CYP) is a synthetic pyrethroid commonly used both outdoor and indoor and is widely reported to have a strong negative impact on sperm motility and count in both animals and humans (4, 13). CYP is known to behave as an inhibitor of protein phosphatase type 2B (PP2B), which has a role in the sperm maturation process (14). Hence, its potential effects on sperm motion made it a valuable choice for this research.

Nowadays, semen analysis is conducted, for both humans and animals, by a trained operator following a manual approach. The traditional method is based on the determination of both macroscopic and microscopic features of sperm, mainly focusing on volume, density, initial motility and concentration (15, 16). Assessing sperm healthy state is crucial prior to artificial insemination to ensure high semen quality for successful fertilization. It also helps to study the impact of external factors like toxins and pesticides on animal fertility. Variables such as age, breed, collection timing, interval, and seasonal variations can influence the quality of bull sperm as well (17). Moreover, many laboratories started to use the computer assisted sperm analysis (CASA) based on different algorithms for the analysis of images or recordings to determine both morphological and kinematic sperm features, respectively (18–21). Among the kinematic parameters, the straight-line velocity (VSL), curvilinear velocity (VCL) and linearity (LIN) are of great interest for the evaluation of sperm progressiveness. The latter, indeed, has been associate to a good fertilization outcome for both humans and bull sperm (22, 23). Several commercially available systems – such as Hamilton-Thorne IVOS® (Beverly, MA, United States) and the Sperm Class Analyser (SCA®) (Microptic Automatic Diagnostic Systems SL, Barcelona, Spain) – are employed in the evaluation of fertility for both human and animals (24, 25). These systems allow an automatic acquisition and classification of sperm based on their motility and morphology. It has been found that these systems can reduce but not completely exclude the operator-dependent variables (26). However, they employ standard condition for the acquisition.

Regarding the acquisition chambers, it is recommended to use a depth of 10 – 20 *μm* which is considered optimal for motility evaluation (27). However, such confined geometry could limit the observation of the native three-dimensional (3D) motility of sperm, confining their motion vertically, given that sperm head is ~4−5μm wide and the flagellum is ~40−50μm long (28, 29). To date, also deeper chambers - having a depth of 100 *μm* - have been developed to evaluate possible azoospermia condition which requires a larger volume to accurately assess sperm count (30). Moreover, motility evaluation in deeper chambers has been reported in previous works, where significantly different patterns were observed with respect to standard observation chambers (31, 32). Hence, as sperm exhibit various complex 3D motility patterns, using unconfined geometries with greater depth could provide more detailed insights into their behavior (33).

In addition, conventional systems generally allow recordings of 1s , limiting the possibility to reconstruct longer trajectories (i.e., circular paths) described in condition of high spatial confinement at the fluid-wall interface (34). It has been assessed the importance of circular trajectories since they could be related to the physiological movement of sperm in response to the surrounding fluid properties, like increased viscosity and the presence of a chemoattractant (35–37). Indeed, in presence of a gradient, sperm move in a diffusive manner toward the higher concentration drifting the circular path due to a change in internal calcium ion concentration (38). Longer trajectories reconstruction owing to longer recording time could be useful to reconstruct this drifting behavior which occurs at long times. In addition to the expensive commercial CASA systems, new open-access, free and easy to use software were developed (39–43).

Here, we present a proof of concept for precise bovine sperm motility analysis in unconfined conditions, acquiring videos of tunable duration at different frame rates. Analyzing sperm motion in different contexts allows to retrieve additional information on sperm motility, enlarging the dataset already provided by standard semen analysis. For the acquisition a glass capillary of 200μm in depth was chosen, as they are usually used in our research group. Moreover, the analysis has been performed by means of a self-written MATLAB tracking routine which reconstructs sperm trajectories, calculates VSL, VCL and LIN and classifies them in sub-populations, based on fixed VCL cut-offs. Other kinematic parameters such as the average path velocity (VAP), beat-cross frequency (BCF) and amplitude of lateral head displacement (ALH) were not evaluated due to the lack of standardization for the sperm average path computation, which limit the comparability among different CASA algorithms (44). Physiological sperm motility was investigated, where only time affected their motility versus altered one due to CYP drug interaction. Until now, CYP has only been used to assess its cellular toxicity at concentrations between 1 to 64μM (45, 46) in rats and at 10μM in humans (47), which are much higher than the concentration needed to inhibit PP2B (48). The chosen concentration of CYP, close to the inhibitory concentration (0.1 nM), may not exhibit toxicity but could potentially induce an increase in sperm motility and progression since CYP is an inhibitor of PP2B. Such increased progression could translate into a greater success in oocyte fertilization. The unconfined conditions ensure that sperm motility remains unhindered by wall influences, as the characteristic dimensions of sperm are much smaller than those of the capillaries used in our experiments. Along with this, the extended acquisition time facilitates the identification of modified swimming patterns, such as circular trajectories, and allows for the evaluation of curvature radius (R). Additionally, our adjustable acquisition and analysis parameters accommodate the requirements of various experiments, enabling analysis of sperm motility for research purposes. The flexibility in acquisition duration and frame rate adjustment allows for the reconstruction of swimming patterns under different microenvironmental and experimental conditions.

To conclude, the aim is to propose an additional versatile sperm motility analysis to characterize sperm behavior in unconfined geometry to better replicate the 3D environment. Moreover, to demonstrate the versatility of this approach, the CYP addition was useful to induce alterations in progressivity and motion patterns that were successfully captured.

## 2 Materials and methods

### 2.1 Sample preparation

Cryopreserved bovine sperm were purchased in frozen straws from ABC Love Genetix (CONSWORK srl, Lodi, Italy) and stored in liquid nitrogen until the experiment. To reduce data biases due to possible variations of different animal characteristics and health status, all the experiments were performed by employing sperm from the same bull, whose data are reported in Table 1. One experimental replicate was performed *per* experiment. Two straws were used, one for each condition. Each straw contained 250μl of volume at a concentration of $30\;x\;10^{6}\;\mathrm{sperm}/\mathrm{ml}$ . For each experiment, a straw was retrieved from the liquid nitrogen container and left in an oven at 37*°C* in a water bath for 1min to defreeze. Afterwards, spermatozoa were washed 3 times using phosphate-buffered saline (PBS) solution and isolated from seminal plasma by centrifugation ( 500xg for 5min at 21*°C*). After the removal of the supernatant at the end of the last centrifuge, the pellet was re-suspended in the Sperm Preparation Medium (Origio, Copenhagen, Denmark) to a final sample volume of 100μl at a concentration of $30\;x\;10^{6}\;\mathrm{sperm}/\mathrm{ml}$ . Although this medium is not commonly used for bovine sperm, it was employed for research group needs. Furthermore, it is equivalent to the more widely adopted SP-TALP medium, except for the following ingredients which have analogous function: glucose, human albumin solution, magnesium sulfate and synthetic serum replacement (SSR). Cypermethrin (CYP) (ML-PR100-0050) was purchased from Enzo Life Sciences Inc. (Farmingdale, New York, United States) and it was reconstituted in solution according to the instructions of the manufacturer by dissolving it in ethanol 100% . Two different samples were used for the two tested conditions: one for the control (CTRL), in which no inhibitor was added, and another for CYP condition $\left(0.1\;nM\right)$ . Both samples were incubated at 37*°C* in a 5% CO_2_ environment. For CTRL, 10μl of sample was diluted in 190μl of sperm preparation medium, while for CYP condition, 10μl of sample was diluted in 190μl of the CYP solution. Sperm motility was assessed for three time-steps: 10 , 30 and 60min .

**Table 1:** Bull characteristics.

Age	2 years
Weight (dead)	470 Kg
Reproductive history	137 offspring

### 2.2 Viability assay

The CellTiter 96® AQueous Non-Radioactive Cell Proliferation Assay (Promega, Madison, Wisconsin, United States) was used to assess viability in cryopreserved bovine spermatozoa. In details, $7.5\;x\;10^{6}$ spermatozoa were mixed with 20μl of CellTiter 96® to a final sample volume of 100μL , following 1h incubation at 37*°C*. Using the Infinite® 200 PRO (TECAN, Genius, Männedorf, Switzerland), absorbance was measured at 490nm . A dependable and exacting method of evaluating spermatozoa vitality has historically been the reduction of tetrazolium compounds (49).

### 2.3 Acquisitions

The optical setup includes an inverted microscope (X81, OLYMPUS) and a CMOS camera (ORCA flash 4.0, Hamamatsu Photonics K.K.) with a 20X brightfield objective. The sperm samples were loaded in a 0.2x2mm glass capillary ( ~1$ each) with rectangular section (CM Scientific) by means of capillarity, and the two extremities were sealed with a capillary wax (Vitrex Medical A/S, Denmark) to avoid drifting of the sample liquid. 10s recordings was performed at a frame rate of 100fps , using an exposure time of 3ms and were repeated three times, at each experimental time-step. The frame rate enabled the reconstruction of sperm trajectories with greater detail. This was achieved through a higher acquisition frequency compared to traditional methods. Additionally, using a deeper chamber for acquisition prevented any hindrance to sperm movement, given that it is approximately three times larger than the average size of bull sperm.

### 2.4 Analysis

#### 2.4.1 Sperm head tracking

Single sperm heads were tracked with a self-written MATLAB routine (version 2022b). Briefly, an initial image filtering procedure (Gaussian filter, morphological opening, mean subtraction and Wiener filter in order) is performed to enhance the heads with respect to the surroundings (50). The embedded MATLAB function ‘*imfindcircles*’, set with a sensitivity S comprised between 0 and 1, detects the apparent head centroid (AC) coordinates, which is the brightest point in the sperm head area after the filtering process. The sensitivity factor is directly proportional to the probability of false detections. For linking, our software uses a Hungarian-based algorithm, by virtue of which a link is created between ACs found to be the closest within a maximum distance D , ensuring the minimization of the sum of pair distances among all ACs between two consecutive frames (51). The algorithm is also endowed with a feature that deals with tracking gaps, when an AC in one frame is not detected in the following one. Such missing data points should be avoided to minimize track breaks or incorrect linking. After the linking step, a second iteration is done through the data for gap-closing, that is investigating track ends to check for possible track interruption due to missed detections. If a track starting point is found close to the endpoint of a subsequent one within a distance $D_{\mathrm{gap}\;\mathrm{closing}}$ , a link spanning at most a desired number of frames G can be created restoring the track. The gap-closing step uses the nearest-neighbor algorithm which is based on a local distance optimisation: the two closest ACs among two tracks are sought for first, then the second closest pair, excluding the first, etc., ensuring that the resulting linking will not depend on the order of the AC in each set. For the tracking, the maximum linking distance D was set equal to 5μm *ca.* A strategy to rule out possible errors due to gap closing procedure was implemented, consisting in computing a mean distance between all consecutive points and check if there are two points along the track whose mutual distance is 5 times the mean one. This value was chosen through a trial-and-error process. In this case, the trajectory is split in two for motility evaluation. A threshold can be set on both track length and VSL to remove noise. The resulting velocities are converted from pixels to microns thanks to the pixel size, which depends on the camera used for the acquisition. All the aforementioned parameters can be tuned to accommodate for specific experimental needs like noisy field of view and frame rate, which in our case was 100fps to better reconstruct sperm motility patterns. The parameters used for the analysis are summarized in Table 2.

**Table 2:** Set of acquisition parameters used for the analysis.

Acquisition parameters	
Frame rate	100fps
Pixel size	6.5 *μm*/*pixel*
Centroid location function sensitivity S	0.6
Gap closing frame interval G	5 frames
Gap closing distance $D_{\mathrm{gap}\;\mathrm{closing}}$	8.5 *μm*
Max linking distance D	4.2 *μm*
Minimum track length	100 points
Minimum VSL	2 *μm*/sec

#### 2.4.2 Motility parameters computation and classification

Once the tracks are evaluated, VSL, VCL, LIN were computed according to the definitions of CASA terminology (52) and formulation of other open-access CASA softwares (39, 43). The equations and definitions implemented in our tracking routine are reported in Table 3. Moreover, the curvature radius R of the sperm trajectory was evaluated with a circular fitting based on the Pratt method, accounting for sperms swimming in a circular mode (53).

**Table 3:** Definition of implemented kinematic parameters.

Parameter	Definition	Computation
VSL	Straight-line distance between first and last points of the trajectory, corrected for time.	$d\left(P_{\;1},P_{N}\right)*\frac{FR}{N-1}*\gamma$
VCL	Distance traveled by the sperm along its curvilinear path, corrected for time.	$\left[\overset{N-1}{\underset{i=1}{\sum }}d\left(P_{i},P_{i+1}\right)\right]*\frac{FR}{N-1}*\gamma$
LIN	Comparison of the straight-line and curvilinear path. It expresses the relationship between the 3D path and the net space gain of the cell.	$\frac{VSL}{VCL}*100$

$P_{i}$ is the i−th point of a trajectory of length N ; $d\left(P,Q\right)$ is the Euclidean distance between point P and Q ; FR is the frame rate; γ is the conversion factor from pixel to microns.

Sperm were classified in motility classes according to the criterion used in SCA® instruments, specific for bovine sperm and shown in Table 4 (54). To remove immotile cells, a minimum threshold for LIN equal to 10% was set since it has been verified that this threshold cuts out immotile cells and noise deriving from out-of-focus objects.

**Table 4:** Motility class definitions according to literature (54).

Class	VCL [*μm* sec^−1^]
Slow progressive	25<VCL<80
Medium progressive	80<VCL<150
Rapid progressive	VCL>150

Tracking results are presented in terms of mean velocity for each motility class at the three different time-steps; furthermore, the numerosity variation for each class among consecutive time-steps is evaluated as a percentage variation according to the following equations:


(1) $$\%\mathrm{variation}\left(30vs10\right)=\frac{\left({\%}_{{\mathrm{Treatment}}_{30\;\min}}-{\%}_{{\mathrm{Treatment}}_{10\;\min}}\right)}{{\%}_{{\mathrm{Treatment}}_{10\;\min}}}*100$$

(2) $$\%\mathrm{variation}\left(60vs30\right)=\frac{\left({\%}_{{\mathrm{Treatment}}_{60\;\min}}-{\%}_{{\mathrm{Treatment}}_{30\;\min}}\right)}{{\%}_{{\mathrm{Treatment}}_{30\;\min}}}*100$$

Equation 1 describes the calculation of the class percentage variation between 30min and 10min while Equation 2 describes the variation between 60min and 30min in order to show how the percentage of sperm falling in each one of the three classes changes during time for each condition. In both equations, the quantities ${\%}_{\mathrm{Treatmen}t_{10\;\min}}$ , ${\%}_{\mathrm{Treatmen}t_{30\;\min}}$ and ${\%}_{\mathrm{Treatmen}t_{60\;\min}}$ represent the percentage of sperm in a specific class and for a specific condition at 10 , 30 and 60min , respectively.

#### 2.4.3 Statistical analysis

The motility data are reported as mean value with standard deviation. Due to non-normality of data distribution the statistical analysis was performed with a pairwise Kruskal-wallis analysis due to non-normal distribution of data (55). The MATLAB function ‘*kruskalwallis*’ was employed (Supplementary Tables S1, S2). The comparison was done for each parameter of a specific class between time-steps of the same experimental condition. Experiments of CTRL and CYP were not compared between each other since they were realized starting from two different bovine samples.

## 3 Results

**Figure 1:**
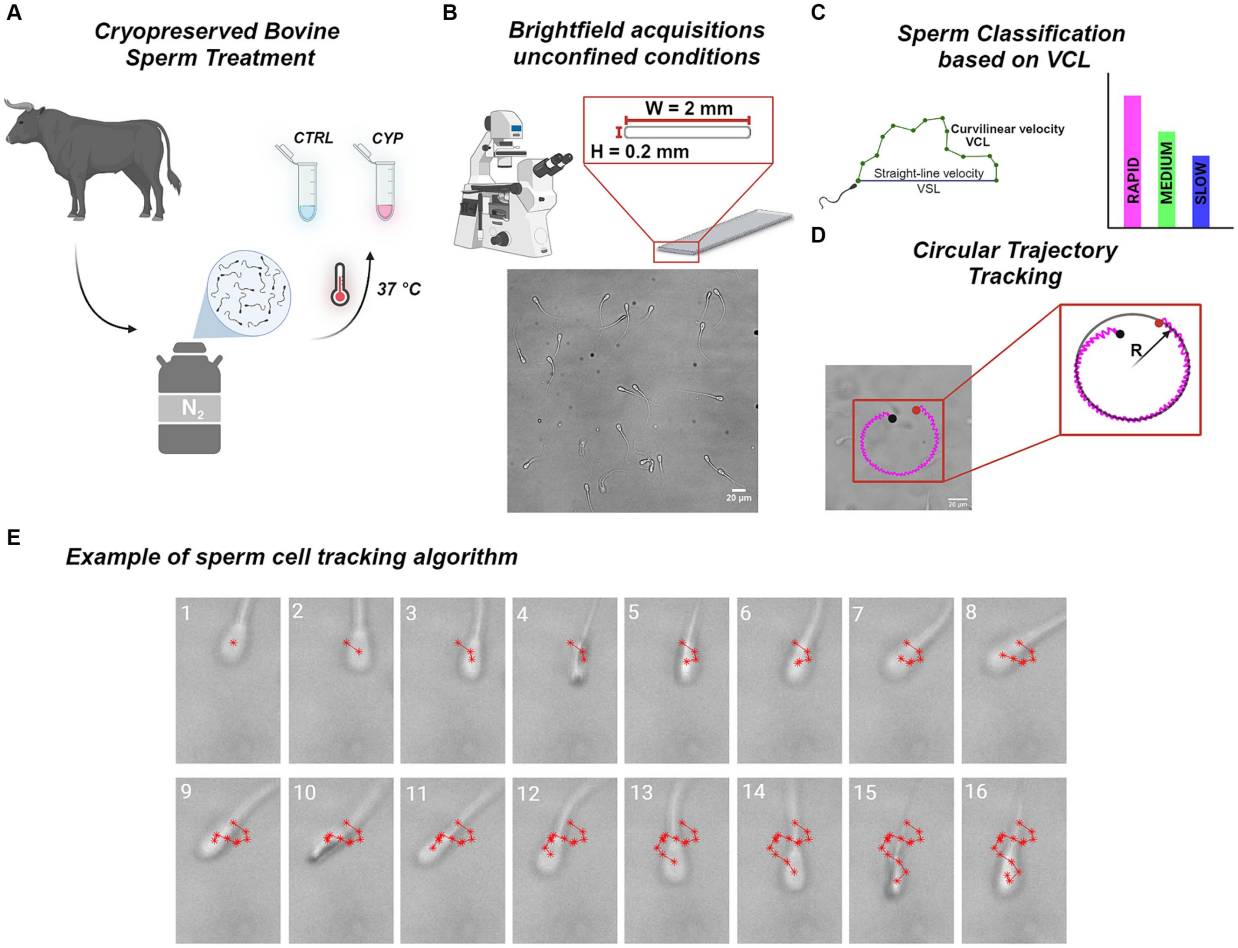
Experimental workflow. **(A)** Cryopreserved sperm from a single bovine was thawed at 37*°C* and an aliquot were treated with CYP at a concentration of 0.1nM , while another was incubated as a control condition. **(B)** Schematic representation of the glass rectangular capillary $\left(0.2x2\;\mathrm{mm}\right)$ in which acquisitions were performed. On the bottom, an example of a brightfield recoding is shown. **(C)** Sperm were tracked by our self-written tracking routine and their velocity were extracted starting from the reconstructed trajectories. In details, we calculated VSL, VCL and their ratio, LIN. The VCL was used to classify sperm in three classes: Slow, Medium and Rapid as in (54). The column graph on the right is only a sketch of the classification in which different colors encode for different sub-classes: magenta for Rapid, green for Medium and blue for Slow. **(D)** Tracking of circular trajectories described by specific sperm. On the left there is a plot of the circular trajectory on top of a brightfield acquisition. On the right a schematic representation of the circular fitting used to evaluate the radius of curvature (R) of the detected track. The black and red dot represents the starting and end points of the trajectory, respectively. The magenta color of the track indicates that it is a rapid sperm cell. **(E)** Example of the tracking process employed by the tracking routine: a sperm cell is tracked frame by frame along the brightfield acquisition locating the AC (denoted by the red *) and then connecting all the detected points to reconstruct the trajectory. The gap closing procedure allows to restore the track bridging ACs missing detections, as it can be seen from frame 3 to frame 5. Each image represents a single frame distinguished by the white number on the top indicating the order.

Bovine sperm motility was analyzed to investigate only the effect of time as control condition (CTRL) and the interplay of these factor with the action of CYP (Figure 1A). CYP works as an inhibitor of PP2B, one of the main regulators of sperm motility. At physiological condition, while sperm undergo the maturation process in the epididymis until becoming fully motile, PP2B activity is progressively being inhibited (14). Acquisitions were performed in unconfined conditions (Figure 1B) which do not confine sperm physiological motion. VCL was used to classify sperm in sub-populations (Table 3; Figure 1C) and altered swimming patterns such as circular trajectories were reconstructed by calculating the curvature radius (R) (Figure 1D). Brightfield recordings were analyzed with a self-written tracking routine to evaluate the motility parameters related to sperm progressiveness, namely VSL, VCL and LIN. The tracking process is based on the detection of the ACs in each consecutive frame of the recording which are in turn connected for the trajectory reconstruction (Figure 1E). Thanks to the gap closing procedure, it was possible to restore the track in case of missed detection as happens between frame 3 and 5, where the AC is not detected due to the head illumination during the rim-on phase in frame 4 (Figure 1E).

**Figure 2:**
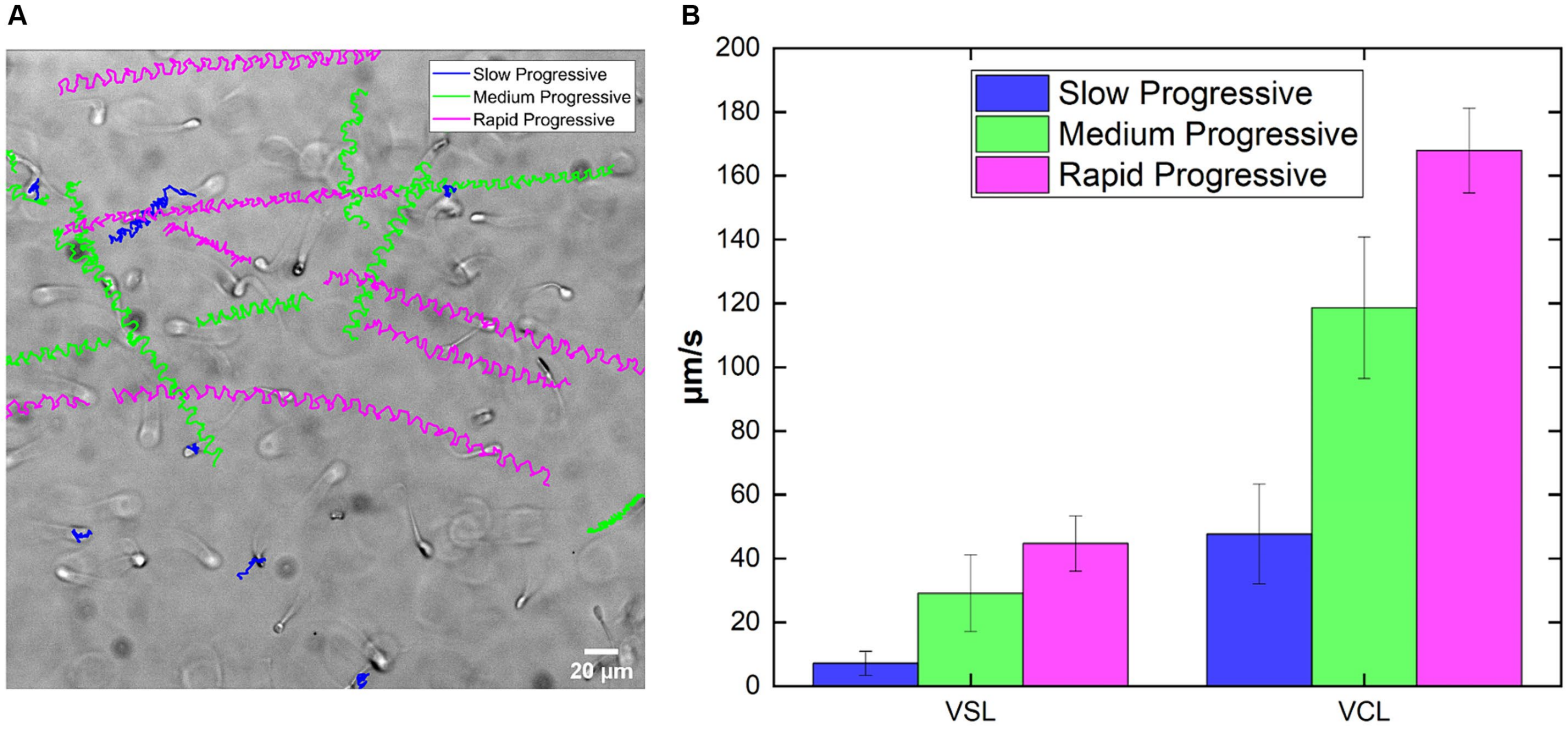
Output of the analysis and set of parameters for the acquisition. **(A)** Example of a field of the 20X acquisition of sperm at CTRL showing the trajectories reconstructed along the movie, color-coded for motility class: blue for Slow, green for Medium and magenta for Rapid. **(B)** Example of the output in terms VSL and VCL, calculated starting from the trajectories of sperm tracked in the movie in **(A)** $\left(N_{\mathrm{Slow}}=15,N_{\mathrm{Medium}}=19,N_{\mathrm{Rapid}}=15\right)\text{.}$

A brightfield acquisition of sperm swimming inside the rectangular glass capillary in the CTRL is shown ( 10min ). On the image there are the reconstructed trajectories obtained by tracking single sperm in the field of view and following them during the whole recording (Figure 2A). Different colors indicate different motility classes, each one related to specific ranges of VCL (Figure 2B; Table 3). From the routine output, the slow class are the most different among the three, while medium and rapid progressive are closer in terms of mean values, both for VSL and VCL. In details, there is a gradual increase among the classes going from slow to rapid classes in both VSL and VCL case (Figure 2B). Our motility values are comparable to those of cryopreserved bovine sperm velocities found in literature, even if acquired at different frame rate and in different confinement (56, 57). In addition, sperm motility was analyzed in capillaries with varying depths and at different frame rates to demonstrate the versatility of the approach (Supplementary Figure S1).

**Figure 3:**
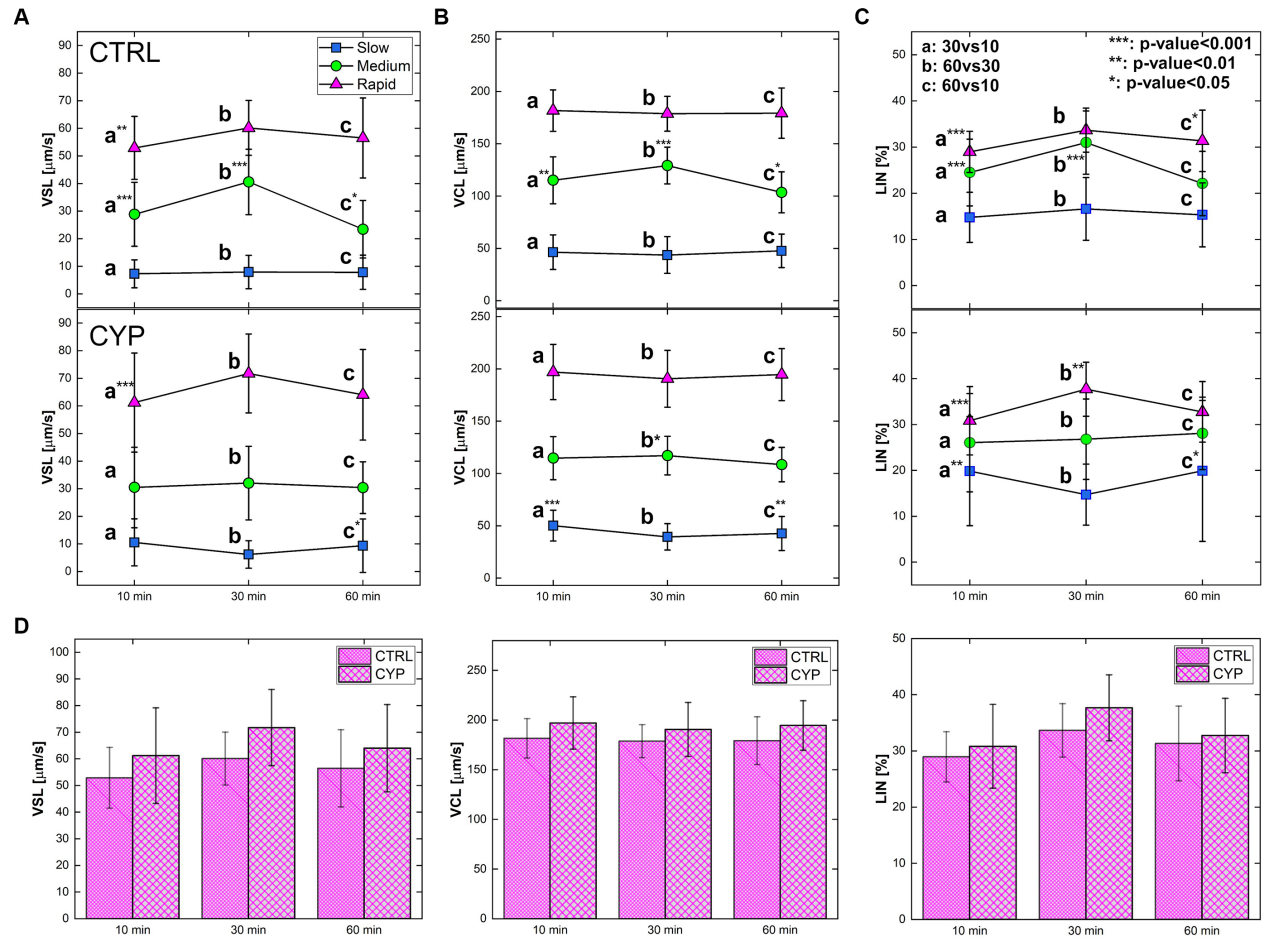
Motility parameter of sperm in both CTR and CYP for the three sub-populations and at the three time-steps. **(A)** VSL, **(B)** VCL **(C)** LIN at three timesteps for both CTRL (on the top) and CYP (on the bottom). Each plot shows the motility parameter associated with each condition, for all the sperm classes encoded by different colors: blue for slow, green for medium and magenta for rapid. The statistical analysis has been performed for each parameter of a specific class between time-steps of the same experimental condition: 30vs10, 60vs30 and 60vs10. The sub-populations were not compared among each other. For each sub-population, a: 30vs10, b: 60vs30 and c: 60vs10. The degree of significance – based on *p*-value – is represented by the * associated to the letters (see also Supplementary Tables S1, S2). ***p-value<0.001, **p-value<0.01 and *p-value<0.05. **(D)** VCL, VSL and LIN of the rapid class of both CTRL and CYP at the three time-steps. Statistical analysis has not been performed between CTRL and CYP since two different samples were employed.

Motility analysis on sperm along the three time-steps are reported in terms of VSL, VCL and LIN for both CTRL and CYP (Figure 3; Supplementary Movies S1–S6). Sperm viability after CYP treatment was assessed at 10, 30 and 60 min, where the percentage of viable cells was ~102%,~103% and ~109% respectively after normalization to the CTRL condition. It was observed that, for both VSL and VCL, rapid sperm are faster at CYP compared to CTRL. Indeed, at 10min , VSL and VCL at CYP are 16% and 8% higher than CTRL, respectively (Figures 3A,B). At 30min the increase is of 19% and 7% , while at 60min the increase is 13% and 9% . Looking at the single classes, the VSL of rapid sperm increases between 10 and 30min for both conditions (Figure 3A) and that these variations are statistically different ( p<0.01 for CTR and p<0.001 for CYP - Supplementary Tables S1, S2). Moreover, this increase is more pronounced in CYP. An increasing progressiveness along time is reflected in the increment in LIN ( p<0.001 ; Figure 3C) since VCL remains almost constant between the two time-steps. The variation of the kinematic parameters of the rapid class at CYP with respect to CTRL can be further appreciated in Figure 3D. Instead, the medium class progressiveness (LIN) increases at CTRL ( p<0.001 ) being more influenced by time (Figure 3C–top). Conversely, the slow sperm class is less influenced by both incubation and treatment effect since the values of VSL, VCL and LIN remain constant for the whole duration of the experiment, except for the decay in LIN observed at 30min ( p<0.01 ; Figure 3C – bottom). An opposite change in the parameters can be observed by looking at the 60min condition in which both velocities for the rapid sperm undergo a decrease at both CTR and CYP, which is stronger for VSL, leading to a decay of LIN (Figure 3C). An exception to this is the medium class with a constant VSL and LIN at 60min for the CYP condition (Figure 3C – bottom). The variations in VSL at CYP, more relevant with respect to VCL (Figure 3B), are reflected in the variation of LIN (Figure 3C), which increases at 30min for the rapid class ( p<0.001 ) and decreases for the slow class ( p<0.01 ), remaining invariant for the medium class (Figure 3B – bottom).

**Figure 4:**
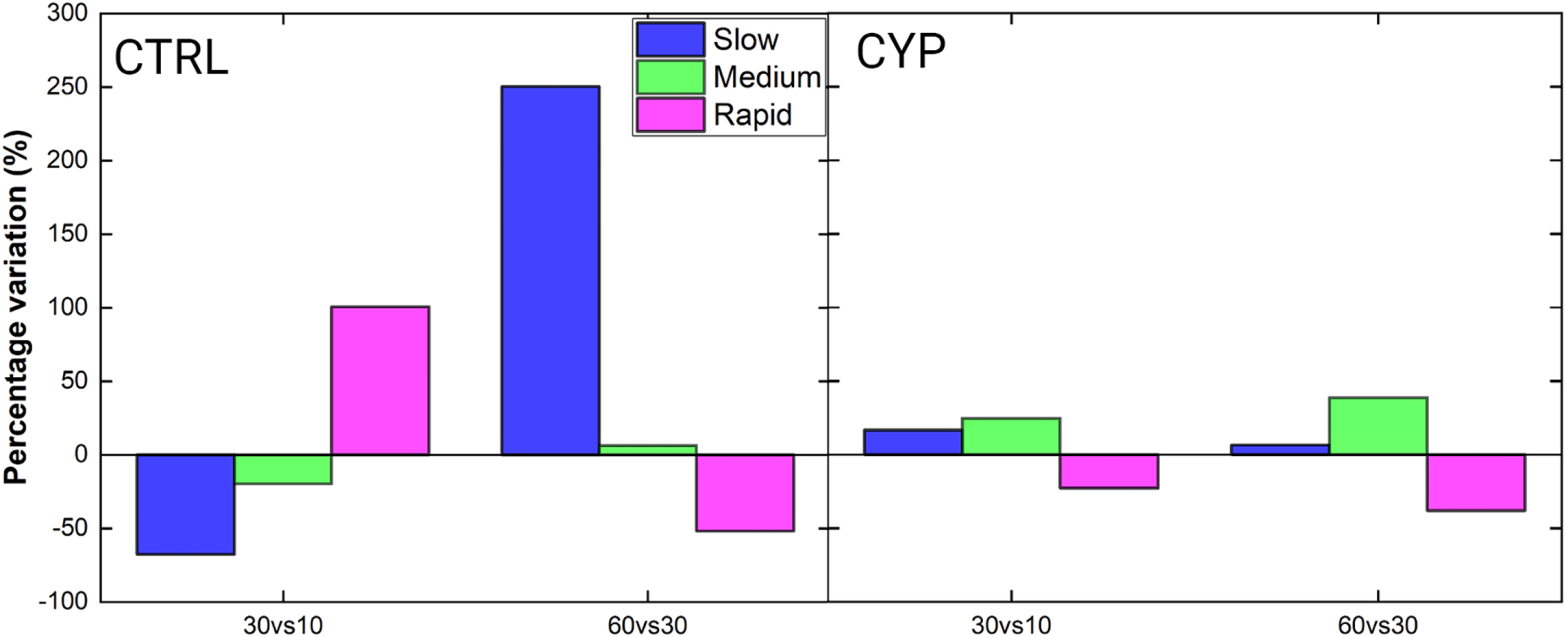
Percentage variation of sperm sub-populations, color encoded as described in the legend, between time-steps. The 30min condition was compared to the 10min one while the 60min one was compared to the 30min by means of Equations (1, 2). A positive percentage variation indicates an increase in the number of sperm falling in a specific class, while a negative variation indicates a decrease with respect to compared condition. The total number of the acquired motile cells are, for the CTRL, $N_{10\;\min}=124$ ( ${N_{\mathrm{Video}\;}}_{1}=49\text{,}$ ${N_{\mathrm{Video}\;}}_{2}=30\text{,}$ ${N_{\mathrm{Video}\;}}_{3}=45)\text{,}$ $N_{30\min}=134$ ( ${N_{\mathrm{Video}\;}}_{1}=75\text{,}$ ${N_{\mathrm{Video}\;}}_{2}=15\text{,}$ ${N_{\mathrm{Video}\;}}_{3}=44)\text{,}$ ${N_{60}}_{\;\min}=126$ ( ${N_{\mathrm{Video}\;}}_{1}=32\text{,}$ ${N_{\mathrm{Video}\;}}_{2}=43\text{,}$ ${N_{\mathrm{Video}\;}}_{3}=51)\text{,}$ while for CYP $N_{10\min}=672$ ( ${N_{\mathrm{Video}\;}}_{1}=246\text{,}$ ${N_{\mathrm{Video}\;}}_{2}=191\text{,}$ ${N_{\mathrm{Video}\;}}_{3}=235)\text{,}$ $N_{30\min}=129$ ( ${N_{\mathrm{Video}\;}}_{1}=43\text{,}$ ${N_{\mathrm{Video}\;}}_{2}=55\text{,}$ ${N_{\mathrm{Video}\;}}_{3}=31)\text{,}$ $N_{60\min}=93$ ( ${N_{\mathrm{Video}\;}}_{1}=33\text{,}$ ${N_{\mathrm{Video}\;}}_{2}=33\text{,}$ ${N_{\mathrm{Video}\;}}_{3}=27)\text{.}$

Motility classes were evaluated and a percentage of variation of each one was calculated and implemented as in Equations 1,2. The results are shown for both CTRL and CYP showing the percentage variation of each class referred to the compared conditions, i.e., 30min versus 10min and 60min versus 30min (Figure 4). At CTRL, going from 10 to 30min , there is a strong decrease of the number of slow sperm and an even stronger increase of the rapid ones, while the medium also decreases. Here the increment in the percentage of rapid sperm is in line with the one observed in VSL (Figures 3A,D). The exact opposite occurs at 60min where there is an increase of the slow sperm by the 250% , along with a less decay of the rapid ones, reflecting what occurs also in terms of VSL and LIN (Figures 3A,C). On the other hand, at CYP the rapid class decline in number- both at 30 and at 60min - compared to 10 and 30min , respectively. However, at 30min , the velocity increases with respect to the starting time. The medium class percentage increments both at 30 and 60min where their VSL remains constant for all times (Figure 3A – bottom), but the LIN slightly increase with time, owing to a decrease of VCL. At 30 and 60min , the rapid class decreases.

**Figure 5:**
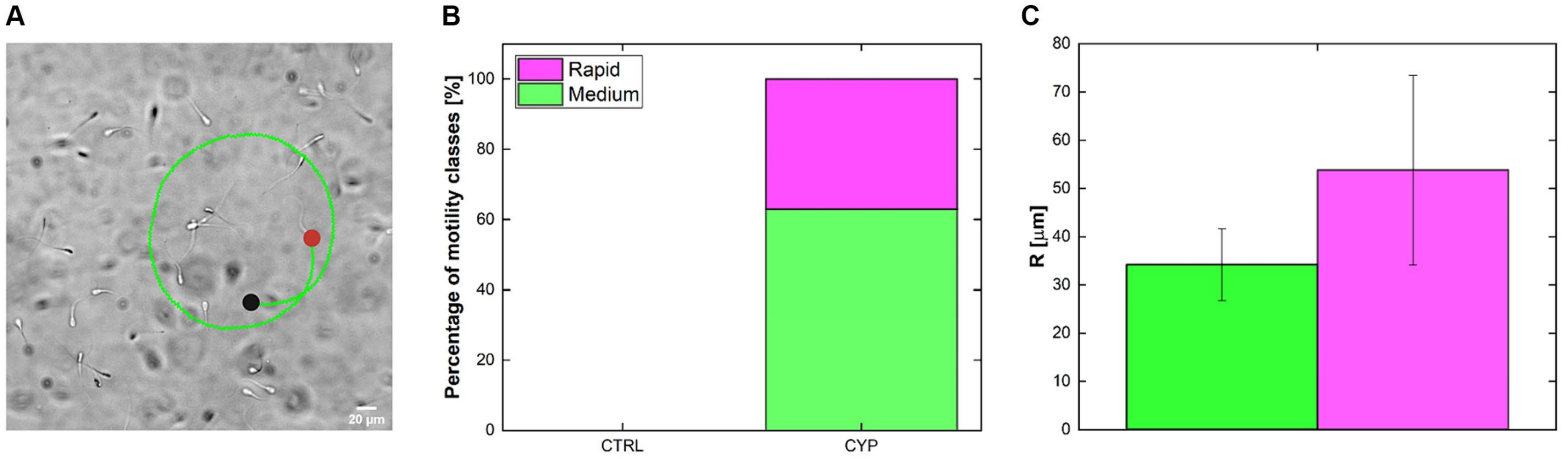
Circular trajectories tracking. **(A)** 20X brightfield acquisition of sperm swimming in the rectangular glass capillary with on the trajectory of a circular swimming sperm. Green color indicated that it belongs to the medium sperm class. The black and red dot represents the starting and end points of the trajectory, respectively. **(B)** Percentage of motility classes to which sperm going in circle belong. No circles were detected in the CTRL condition. In CYP the total $\left(N=16\right)$ is divided between the rapid $\left(\sim 40\%\right)$ and medium $\left(\sim 60\%\right)$ classes and were present mainly at 10min and 30min. **(C)** Circular trajectories radius (R) $\left[\mathrm{\mu m}\right]$ for medium and rapid class. Both the percentages and R are not divided by time.

Besides calculating sperm motility parameters and classifying them in sub-classes, our tracking routine detect altered trajectories such as circular motion. Therefore, the investigation and manual quantification of the presence of sperm moving in circles was performed (Figure 5). The idea was to understand the ability of CYP to influence not only sperm velocity or progressiveness but also the motility patterns. A zoom-in of a brightfield acquisition showing swimming sperm on top an example of a tracked sperm cell moving in a circular mode is shown (Figure 5A). The green color of the trajectory indicates the medium class. There is an evident difference between CTRL and CYP conditions since sperm moving in circle are not present at CTRL (Figure 5B). Instead, the stacked column bars indicate that in CYP a portion of active sperm are induced to move in circles and lose their progressiveness. Of the total number of tracked sperm, ~60% belong to the medium class while the remaining ~40% is rapid (Figure 5B). All the detected circles were found only at 10 and 30min . By performing a circular fitting on the reconstructed trajectories, it was possible to extract information about R (Figure 5C) which changes significantly among medium and rapid sperm ( p<0.05 ) but not between time-steps. Circular trajectories undergo an increase between 10 and 30min of ~29% , calculated by means of Equation 1.

## 4 Discussion

In this work a versatile approach to investigate sperm cell motility in unconfined conditions was proposed. The aim was to provide additional information on sperm motility in contexts different from the standard semen analysis, supporting the CASA system outcome. As a matter of fact, automated analysis -like artificial intelligence (AI) approaches- is becoming increasingly popular in fertility research (58). Therefore, having a larger dataset of descriptive sperm parameters could be beneficial to further characterize sperm behavior. To prove the approach, semen coming from only one bovine was used to avoid biases due to inherent differences among animals such as age and weight (17, 59, 60). By using an easy to use and low-cost glass capillary 200μm deep, recordings were performed at 100fps to reconstruct sperm motility. Single cell tracking and velocity evaluation were performed with a self-written MATLAB routine to extrapolate VSL, VCL and LIN and retrieve sub-populations of sperm (rapid, medium and slow). Despite the relevance of other kinematic quantities like VAP, such parameters were not evaluated due to lack of standardization for the sperm average path calculation. Therefore, classification was performed on VCL cut-offs found in literature (54, 61) The results obtained are in line with the velocity rages found in literature with CASA system (56, 57). Slight differences could be observed mainly for VCL, which is strongly influenced by the frame rate of acquisition (62).

The unconfined conditions of the acquisitions (i.e., capillary depth) avoid the limitation associated with the confinement of sperm motility in smaller depth, resulting in a better reconstruction of the physiological movement with limited influence by the environment. Effects of geometric confinements indicated a positive correlation between capillary depth and velocities (Supplementary Figure S1). Owing to a longer acquisition duration, it was possible to detect altered swimming patterns, as the circular ones. The settings can be tuned for both acquisition and analysis depending on the experimental needs. The frame rate can be varied to reconstruct different types of swimming patterns described in specific microenvironmental conditions. The duration of the acquisitions is not fixed. In addition, our analysis is not performed with a specific dimension of capillary with a fixed geometry, but it can be varied offering the possibility to test sperm motility in confined or unconfined geometries.

The analysis was applied to cryopreserved bovine sperm treated with CYP to assess its impact on motility. One of the key regulators of sperm motility -protein phosphatase type 2B (PP2B) - is inhibited by CYP. In a healthy state, PP2B activity gradually decreases as sperm proceed through the process of maturation from the caput epididymis to the cauda, where they become motile and ready for ejaculation (14, 63, 64). Indeed, the aim was to induce a further reduction of PP2B activity using a prescribed CYP concentration. The analysis showed an increase of progressive motility on the overall sperm population. Despite literature findings, our work suggests a possible positive effect of the used concentration – far below the toxic limit – of CYP on sperm motility. Indeed, such a used concentration enhances the effect of incubation time and temperature alone (CTRL), generally associated with an increase in sperm velocities (46, 65). According to the velocity outcome, the time-window response of CYP is from 10 to 30min , suggesting that the action of the drug occurs before the end of the entire observation time, since an evident reduction of cell velocity occurs at 60min . Moreover, it was found that rapid sperm are influenced more by CYP, showing the highest progressiveness and numerosity increase among all classes (Figures 3A–D; Figure 4). In addition, the increase of mean VSL for the rapid class combined with the decrease of the slow sperm VSL at 30min , suggest that CYP induces a redistribution of sperm that change their velocity moving from one class to another. This rearrangement can be observed in both the CTRL and CYP conditions in terms of rapid and slow sperm. At 60min , the slow sperm number increases at CTRL more significantly than CYP, where medium increments more (Figure 4). Regarding the rapid class a possible reduction of cell energy could occur since they redistribute to the slow class at the CTRL condition and in the medium class at CYP. Moreover, it was possible to detect circular trajectories owing to the longer duration of our acquisitions with respect to commercial CASA systems, which would have tracked this cell only for a short period of time biasing its correct reconstruction. Circular motion has been found to be correlated to fertilization. As a matter of fact, sperm switch from a straight, symmetrical motion to an asymmetrical one, promoting circular motion in the egg’s proximity. This change in behavior, i.e., hyperactivation, helps them thoroughly search the egg’s surroundings, increasing their chances of finding it (66). The circles were mainly detected at 10 and 30min , in line with the CYP time-window. Since our unconfined conditions do not hinder sperm physiological movement, circular trajectories – generally associated with near-wall motion – are more likely favored by the effect of CYP (67).

In conclusion, the motion of bovine sperm under unconfined conditions upon exposure to a low concentration of pesticide was successfully captured. This exposure resulted in a notable increase in progressiveness within the rapid sperm sub-population after a 30-min period. Additionally, prolonged acquisition revealed the formation of distinctive circular patterns. These alterations in sperm motion -under unconfined conditions- are attributed to the influence of CYP. The versatility of our analysis -which permits to modify setting parameters depending on the experimental needs- allows to perform motility analysis of sperm offering the possibility to acquire at variable frame rates, for longer times and in chambers deeper than those generally used. The proposed approach holds promise as a valuable addition to existing diagnostic tools as it has the potential to explore different experimental conditions, offering new insights and enhancing diagnostic precision in the future.

## Data availability statement

The raw data supporting the conclusions of this article will be made available by the authors, without undue reservation.

## Ethics statement

Ethical approval was not required for the studies on animals in accordance with the local legislation and institutional requirements because only commercially available established cell lines were used.

## Author contributions

LFC: Investigation, Methodology, Validation, Writing – original draft. CDC: Investigation, Methodology, Validation, Writing – original draft. MF: Writing – review & editing. AFF: Investigation, Methodology, Validation, Writing – review & editing. MIM: Conceptualization, Writing – review & editing. DD: Conceptualization, Writing – review & editing. FC: Writing – review & editing, Supervision. PAN: Supervision, Writing – review & editing.

## Supplementary material

SUPPLEMENTARY Movie 1. 1 s cut of a CTRL Condition at 10 min

SUPPLEMENTARY Movie 2. 1 s cut of a CTRL Condition at 30 min.

SUPPLEMENTARY Movie 3. 1 s cut of a CTRL Condition at 60 min.

SUPPLEMENTARY Movie 4. 1 s cut of a CYP Condition at 30 min.

SUPPLEMENTARY Movie 5. 1 s cut of a CYP Condition at 30 min.

SUPPLEMENTARY Movie 6. 1 s cut of a CYP Condition at 60 min.
