# Supplementary material for: Exploring altered bovine sperm trajectories by sperm tracking in unconfined conditions
Source: Front Vet Sci. 2024 Apr 2;11:1358440. doi: 10.3389/fvets.2024.1358440 (PMC11019440; doi:10.3389/fvets.2024.1358440)
Supplement: Supplementary file 7 [file Data_Sheet_1.pdf]

Supplementary materials

1 Supplementary Data

Supplementary Table 1: Pairwise Kruskal-wallis analysis for VSL, VCL and LIN of each class between time-steps, 30 min against 10 min, 60 min against 30 min and 60 min against 10 min at CTRL condition. p-value < 0.05 indicates statistical difference. * p-value < 0.05, ** p-value < 0.01, *** p-value < 0.001, NS not significant.

CTRL	VSL			VCL			LIN		
	**Rapid**	**Medium**	**Slow**	**Rapid**	**Medium**	**Slow**	**Rapid**	**Medium**	**Slow**
30 vs 10	**	***	NS	NS	**	NS	***	***	NS
60 vs 30	NS	***	NS	NS	***	NS	NS	***	NS
60 vs 10	NS	*	NS	NS	*	NS	*	NS	NS

Supplementary Table 2: Pairwise Kruskal-wallis analysis for VSL, VCL and LIN of each class between time-steps, 30 min against 10 min, 60 min against 30 min and 60 min against 10 min at CYP condition. p-value < 0.05 indicates statistical difference. * p-value < 0.05, ** p-value < 0.01, *** p-value < 0.001, NS not significant.

CYP	VSL			VCL			LIN		
	**Rapid**	**Medium**	**Slow**	**Rapid**	**Medium**	**Slow**	**Rapid**	**Medium**	**Slow**
30 vs 10	***	NS	NS	NS	NS	***	***	NS	**
60 vs 30	NS	NS	NS	NS	*	NS	**	NS	NS
60 vs 10	NS	NS	*	NS	NS	**	NS	NS	*

2 Supplementary Figures

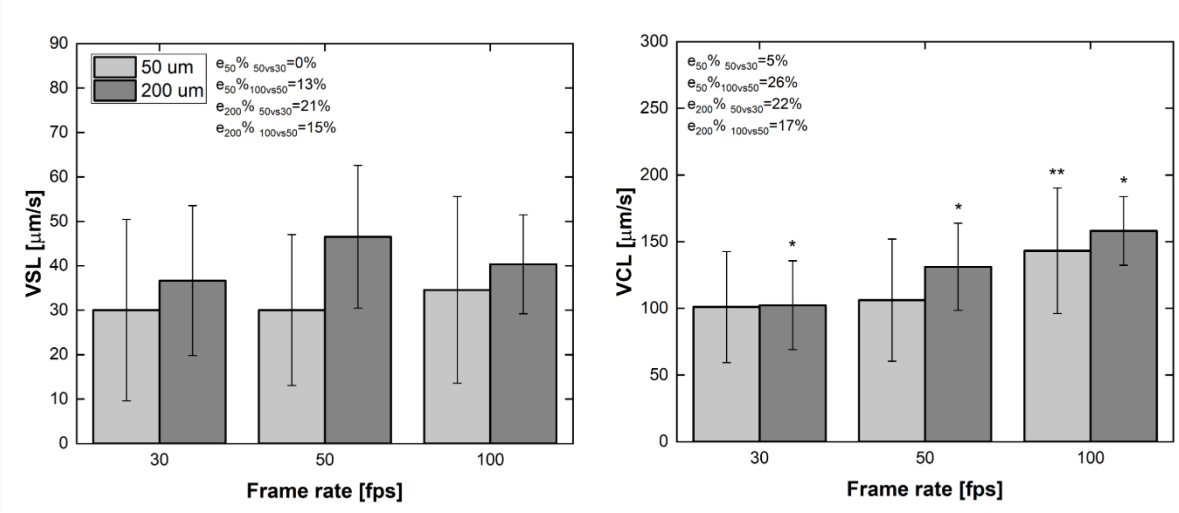

Supplementary Figure 1. Mean VSL and VCL of sperm cells at CTRL (10 min) acquired at three different Frame rates: 30 fps, 50 fps and 100 fps (reported on the horizontal axis) for two capillary depths, 50 μm and 200 μm. Analysis settings, at the frame rate of 30 fps, were: Minimum Track Length: 30 points, Max Linking Distance: 8 μm. At the frame rate of 50 fps, were: Minimum Track Length: 50 points, Max Linking Distance: 6 μm. At the frame rate of 100 fps, were: Minimum Track Length: 100 points, Max Linking Distance: 5 μm (see main text for definition). Cells

with VSL < $2\frac{\mu m}{s}$ and LIN $< 10\%$ were discarded. The error is evaluated between consecutive frame rates for fixed capillary depth and is evaluated as $e_i\% = \left|\frac{V_j + V_{j-1}}{V_j}\right| * 100,$ where i stands for channel depth and j or $j-1$ stand for the actual frame rate and the previous one respectively. At 50 μm, $N_{30} = 21$, $N_{50} = 27$, $N_{100} = 24$. At 200 μm, $N_{30} = 17$, $N_{50} = 17$, $N_{100} = 11$. * p-value < 0.05, ** p-value < 0.01
